# Dr. Theodore F. Chupein

**Published:** 1901-05

**Authors:** 


### Obituary.

### DR. THEODORE F. CHUPEIN.

Dr. Chupein died at his residence, Philadelphia, March 23, 1901. His health had been perceptibly failing for some time, but the end came with startling suddenness to his friends.

Dr. Chupein was born in Charleston, S. C., September 7, 1830. He was educated in the schools of that city, and to this training he added the results of a lifetime of continuous study. This enabled him to grasp with facility the many problems that confronted him in the practice of his profession.

He was placed by his father with Dr. William Stockton Monefeldt, a dentist of reputation in Charleston, who entertained some of the old-time notions that a man to be a good dentist should begin by cultivating manual dexterity, and in order to effect this in the young student he kept him at carving teeth out of ivory blocks for over a year of his novitiate, although that method of preparing artificial dentures had long been obsolete. This apparently useless practice was, however, an important part of his training, for to it, probably, Dr. Chupein owed that mechanical ability that gave character to his work throughout his life. He remained with Dr. Monefeldt for five years as student and assistant, and in 1852 began practice for himself in Charleston.

At the commencement of the Civil War he became a member of the Washington Artillery, of Charleston, and served as sergeant in the Confederate service during the entire period of the conflict, being stationed, at garrison duty, at Adams Run, near Charleston. The commandant at one time being in need of the services of a dentist, and finding Dr. Chupein well qualified, he was selected to act as dental surgeon for the men in the garrison, a duty he fulfilled to the satisfaction of the officers and men, although oftentimes compelled to make his own instruments at the army forge.

During the war his family removed to Philadelphia, and at its close he joined them, obtaining employment with Dr. J. D. White, then the most prominent dentist of that city, and had charge of his mechanical laboratory.

He received the degree of Doctor of Dental Surgery from the Pennsylvania College of Dental Surgery, March, 1872.

He subsequently returned to Charleston, and, in connection with his practice, opened a dental supply house, at that time a great convenience to the dentists of that city. He eventually suffered, in 1875, the entire loss, by fire, of his office and supply department. The following year he again established himself in Philadelphia, where he remained up to the day of his death.

During all this period from 1876 to 1901 he was an untiring worker in the dental ranks of his adopted city. He united at once with the Pennsylvania Association of Dental Surgeons, and subsequently became an active member of the Odontographic Society, since disbanded. He was also a prominent member in the Odontological Society, and was, at a later period, elected an honorary member of the Academy of Stomatology of Philadelphia.

He was made Secretary of the Pennsylvania Association of Dental Surgeons in 1877, and was continued in that office up to the time of his death.

In 1887 he was made editor of *The Dental Office and Laboratory,* published by Johnson & Lund. Dental readers have been made familiar with his practical suggestions upon its pages; in fact, this publication was almost entirely made up from his pen.

He prepared the chapter on "Artificial Dentures on Bases of Fusible Alloys" for the "American System of Dentistry."

In 1890 he published a small work entitled "The Dental Laboratory," full of practical suggestions.

Dr. Chupein lived an active and yet ever an unobtrusive life. He seemed to be almost entirely devoted unselfishly to his profession. The writer rarely ever met one more willing to sacrifice self in the interest of dentistry. His long and varied experience had taught him ways and means to overcome difficulties, and these he was always ready to impart to those needing assistance.

He was thus naturally an ever-faithful worker in the organizations with which he was connected, but his love seemed to be centred in the Pennsylvania Association of Dental Surgeons, and it was largely through his persistent effort that that society managed to tide over a long and almost lifeless period to the present, when it has renewed its youth and now promises something worthy of its earlier life when Townsend, White, Arthur, Neall, and others made it famous among dental organizations. The recrudescence of this organization he was happily permitted to see before he passed away.

Dr. Chupein was honored in his adopted city as a man not only of marked ability in his profession, but one true to his highest conceptions of duty. The motives which actuate some men are at times difficult of solution, but with Dr. Chupein there was no secrecy, no suspicion of an underlying selfish motive. He was always true to those higher principles, as he understood them, which not only made him the good citizen, but the earnest professional worker.

He married, in 1858, Miss Virginia M. Phol, of Philadelphia. He had six children, and a widow and three children survive him.

